# Combined determination of plasma MMP2, MMP9, and TIMP1 improves the non-invasive detection of transitional cell carcinoma of the bladder

**DOI:** 10.1186/1471-2490-6-19

**Published:** 2006-08-10

**Authors:** Andrea Staack, Steffen Badendieck, Dietmar Schnorr, Stefan A Loening, Klaus Jung

**Affiliations:** 1 Department of Urology, University Hospital Charité, CCM, Humboldt University, Berlin, Germany

## Abstract

**Background:**

Matrix metalloproteinases (MMPs) and their inhibitors (TIMPs) play a major role in the maintenance of extracellular matrix homeostasis and are involved in the process of tumour invasion and metastasis in several malignant tumour entities. The goal of this study is to evaluate the diagnostic value of various circulating MMPs and TIMPs in blood plasma for a non-invasive detection of transitional cell carcinoma of the bladder (TCC).

**Methods:**

In this study the concentrations of MMP1, MMP2, MMP3, MMP9, their inhibitors TIMP1, TIMP2, and the MMP1/TIMP1-complex (MTC1) were quantified in blood plasma with the sandwich enzyme-linked immunosorbent assay (ELISA). Blood plasma samples were investigated from 68 patients (non-metastasized, n = 57 and metastasized, n = 11) with TCC of the bladder and from 79 healthy controls. The mROC program was used to calculate the best two- and three- marker combinations. The diagnostic values for all single markers and the marker combinations were estimated both by the overall diagnostic performance index area under the ROC curve (AUC) and the sensitivity and specificity at cutoff limits with the highest diagnostic accuracy and at the 90% and 95% limits of sensitivity and specificity, respectively.

**Results:**

The median MMP2 concentration was elevated in blood plasma in all patient groups with TCC in comparison to the controls (p < 0.001). The concentrations of TIMP1, TIMP2, and MTC1 in plasma probes were significantly lower from patients with non-metastasized TCC compared to the controls. MMP2 tested alone reached the highest sensitivity and specificity at 75%, respectively. The sensitivity and specificity increased when tested in combination with MMP9 and TIMP1 (97%, 94%, respectively). The combination of MMP9 and TIMP1 also showed an improved sensitivity (80%) and specificity (99%) than tested alone.

**Conclusion:**

MMP2 is a statistically significant marker in blood plasma for bladder cancer detection with an increased diagnostic value in combination with MMP9 and TIMP1. This study showed that the highest sensitivities and specificities are not obtained by testing each marker alone. As shown by the best two-marker combination, which includes MMP9 and TIMP1, the optimized combination does not always include the best single markers.

## Background

The transitional cell carcinoma (TCC) of the bladder is the second most common malignancy of all genitourinary tumours after prostate cancer. For the year 2006 the American Cancer Society published an estimated number of approximately 61,420 new cases of bladder cancer in men and of 44,690 new cases in women. According to the American Cancer Society approximately 13,060 men and 8,990 women will die from TCC of the bladder in 2006 in the United States [1]. Hematuria and dysuria are often the only symptoms at an early stage of bladder cancer. Ultrasound, urine analysis, urine cytology, and cystoscopy are currently the most common diagnostic tools. Urine cytology is the "gold standard" for a non-invasive diagnosis, but has a low sensitivity of less then 30% [2]. Cystoscopy with biopsy is an invasive diagnostic method of TCC with so far the highest sensitivity and specificity, >90% respectively [2]. Therefore, it is a compelling task to find a more sensitive and specific non-invasive marker for the early diagnosis of TCC and early detection of recurrences [2-5]. Voided urine is easy and inexpensive to obtain and could be used routinely in clinical practice to look for bladder tumour markers [6,7]. Although voided urine would be ideal for screening, follow-up efforts have been made for bladder tumour detection in blood serum [8,9].

One of the essential alterations that occur in malignancy is tissue invasion and metastasis [10]. Degradation of the basement membrane and the extracellular matrix (ECM) is a prerequisite for tumour invasion. Matrix metalloproteinases (MMPs) belong to the group of ECM degradation enzymes. The balance of secreted MMPs and their specific inhibitors (TIMPs) plays an important role in maintaining connective tissue homeostasis in normal tissue [11]. In neoplastic diseases an imbalance of MMPs and TIMPs, leading to an excess of degradative activity, is supposed to be linked to the invasive character of tumour cells [12,13].

MMPs are divided according to their target protein into several families [14]. In this study we will focus on MMPs, which are described in the literature to have an impact in bladder tumour carcinogenesis: the collagenase MMP1 [15], the stromelysin MMP3 [15], and the gelatinases MMP2 [4] and MMP9 [7,16]. Endogenous proteins known as tissue inhibitors of metalloproteinase (TIMPs) also regulate activities of MMPs [17]. Activated MMP1, MMP3, and latent forms of MMP2 and MMP9 bind to and are inhibited by TIMP1 and TIMP2. Some studies have shown that TIMP1 binds preferably to MMP9 and TIMP2 to MMP2 [18,19].

Studies concerning the expression of MMPs in TCC of the bladder are rare. Few analyzes of MMPs have been conducted in blood serum [20]. Since MMPs and TIMPs are released from platelets and leukocytes into serum during blood collections [21] plasma should be used to determine circulating MMPs and TIMPs [22,23]. To our knowledge there are no data available yet, which determine MMPs, TIMPs, and MMP1/TIMP1-complex (MTC1) in blood plasma as non-invasive tumour markers for TCC. This study is performed to investigate the impact of MMP1, MMP2, MMP3, MMP9, TIMP1, TIMP2, and MTC1 in blood plasma for non-invasive diagnosis of TCC of the bladder.

## Methods

### Patients

A total of 68 patients with TCC of the bladder were included in our study. The study followed the tenets of the Declaration of Helsinki. Our local Medical Ethical Committee (Chairman: Prof. R. Uebelhack, Charite University Hospital, Berlin, Germany) approved the study protocol "Detection of metalloproteinases in patients with genitourinary cancer" on July 16^th^, 2002. From each patient a verbal informed consent was obtained. The tumour was diagnosed by biopsy or obtained by transurethral resection of the bladder (TURB) or radical cystectomy and categorized according to the TNM-classification [24]. Patients were excluded from this study with other diseases or conditions, which are known from the literature to result in an increase of MMPs, e.g. renal cell carcinoma [25], prostate cancer [22], liver diseases [26], rheumatoid diseases [27]. For further staging patients were diagnosed by abdominal ultrasound, intravenous pyelography, computer tomography, magnetic resonance imaging, and/or bone scans additionally to the histopathologically obtained diagnosis. Fifty-seven patients (45 male, 12 female; median age, 68 years, range 42–86 years) with TCC of the bladder (Ta, n = 32; T1/pT1, n = 11; pT2, n = 6; pT3, n = 4; pT4, n = 4; G1, n = 20; G2, n = 23; G3, n = 14) were included in this study without evidence of metastasis. Eleven patients (9 male, 2 female; median age: 61 years, range 39–72) were included in the study suffering from TCC of the bladder (pT2, n = 5; pT3, n = 4; pT4, n = 2) with metastasis. Lymph node metastasis (N1: n = 8; N2: n = 2) was found in 10 patients and organ metastasis in 2 patients.

### Control group

The control group consisted of 40 female (median age: 39 years, range 23–69 years) and 39 male (median age: 48 years, range 20–78 years) healthy volunteers without any history of cancer, inflammatory or immunodeficiency diseases and were tested during the same period.

Laboratory blood tests were performed to determine liver enzymes, prostate specific antigen (PSA), white blood cells, and C-reactive protein. Urinalysis was performed to evaluate urinary infections or kidney diseases. Volunteers with pathological test results were excluded from the study.

### Blood samples

Blood samples were obtained from each patient by venous puncture into lithium-heparin coated blood collection tubes ("monovettes", No. 03.1528, 03.1589 by Sarstedt GmbH, Nuembrecht, Germany) before the beginning of therapy. The blood collection tubes were stored at room temperature (22°C) for 30 min and then centrifuged (1600 × g) at 4°C for 15 min. The supernatant was carefully removed and stored at -80°C until further processing. Samples were thawed at room temperature just prior to the assays.

### Measurement of MMP- and TIMP-concentrations in blood plasma

Plasma MMP- and TIMP-concentrations were measured by using the sandwich ELISA technique as reported previously in detail from our laboratory [28]. We used the commercially available quantitative sandwich ELISA test kits (Amersham International, Little Chalfont, U.K.; article no. RPN 2610, RPN 2617, RPN 2613, RPN 2614, RPN 2611, RPN 2618, RPN 2612) for the determination of MMP1, MMP2, MMP3, MMP9, TIMP1, TIMP2, and MTC1 concentrations in the blood plasma of the patients and the control group. With these ELISA techniques the non-active forms (proforms) of MMPs and in case of MTC1 the complex of MMP1 and TIMP1 were measured. The probes were diluted with assay buffers (MMP1 1:5, MMP2 1:51, MMP3 1:1, MMP9 1:11, TIMP1 1:101, TIMP2 1:4, and MTC1 1:11). The measurements were conducted according to the manufactures guidelines. The cubic-spline method was used for calculation of concentrations (EIA/KIN-star-program, Fa. WEPAH-MED, Berlin, Germany) from the absorbances measured on a microplate reader (HTIII; Anthos Labtec Instruments, Salzburg, Austria) at 450-nm. All samples were measured as duplicates and the mean was calculated for data analysis. A negative control was run for each test as a blank well without plasma probes.

### Statistical analyses

Medians and ranges were calculated for all markers. Non-parametric statistical calculations were performed throughout this study. The Mann-Whitney-U-test for unpaired samples, the Kruskal-Wallis-test for multiple comparisons, and the rank correlation coefficient according to Spearman were used (SPSS 13.01 for Windows, SPSS Inc. Chicago, IL; GraphPad Prism 4.0, GraphPad Software Inc., San Diego, CA). Statistical differences of at least p < 0.05 (two-sided) were considered statistically significant.

ROC analyzes were performed with the program MedCalc, version 8.2.0.1 (MedCalc Software, Mariakerke, Belgium) and with the mROC program, version 1 for combining tumour markers [29]. Because of multiple inferences obtained by ROC analyzes, the p-values were adjusted by the sequentially rejective Bonferroni test [30].

## Results

### Plasma concentrations of MMP1, MMP2, MMP3, MMP9, TIMP1, TIMP2, and MTC1 in TCC of the bladder

In the control group, plasma concentrations of MMP1, MMP2, MMP3, MMP9, TIMP1, TIMP2, and MTC1 are not correlated to age (r_s _= -0.196 to 0.109; p > 0.05) and are not significantly different between female and male controls (Mann-Whitney U-test, p < 0.05). The data were therefore combined and compared with the results of the bladder carcinoma patients.

All results of this study are of exploratory nature and are summarized in Table 1. In the group of patients with non-metastasized TCC of the bladder only the plasma concentration of MMP2 was significantly higher in comparison to the controls. The plasma concentrations of TIMP1, TIMP2, and MTC1 were significantly lower in patients with TCC compared to the control group (Table 1). A more detailed analysis showed that all parameters were not different between patients with superficial (n = 43) and invasive (n = 14) TCC of the bladder (p = 0.258 – 0.767). Considering the effect of histological grading on the MMP and TIMP levels, only MMP1 differed between grade 1 and 2 (median 4.4 vs. 1.9, p = 0.006) while the concentrations of all parameters were independent of the histolological grade (p = 0.101 – 0.532). In metastasized patients, the concentration of MMP2 (p < 0.001) and the ratio of MMP2 to TIMP1 (p < 0.001) were significantly elevated compared to the control group. Plasma concentrations of MMPs, TIMPs, and MTC1 except for TIMP2 were higher in the group of metastasized tumours compared to the patients of non-metastasized TCC (Table 1).

### Correlation of MMPs and their inhibitors between each other and to the TNM-classification

Table 2 presents the Spearman's correlation coefficients among the MMPs, TIMPs, and MTC1 and also the relationships of the markers to the tumour stage and grade. TIMP1 in blood plasma correlated not only to tumour stage and grade (Table 2) but also to the lymph node involvement (r_s _= 0.275; p = 0.027). There was a statistically significant correlation between the MMP2 plasma concentration and distant metastasis of TCC (r_s _= 0.259; p = 0.035). Plasma concentrations of MMP1, MMP3, MMP9, TIMP2, and MTC1 did not correlate to tumour stage and grade (Table 2), but also to lymph node involvement or distant metastasis (data not indicated).

### Diagnostic validity of the single markers and marker combinations

**Figure 1:**
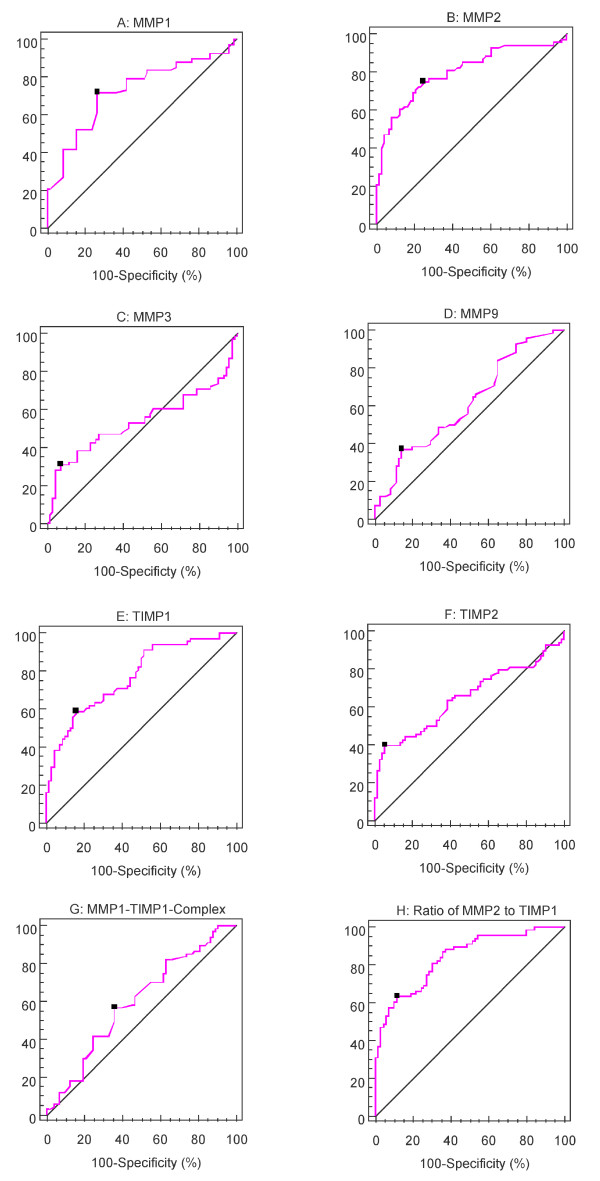
A-H. ROC curves for MMP1, MMP2, MMP3, MMP9, TIMP1, TIMP2, MMP1/TIMP1-complex, and the ratio of MMP2 to TIMP1 from bladder cancer patients. The healthy controls are included. AUCs are given in Table 2. MMP2 showed the largest, MMP3 the highest AUC. The cutoff point (black square box) in each curve indicates the point with the highest diagnostic accuracy.

**Figure 2:**
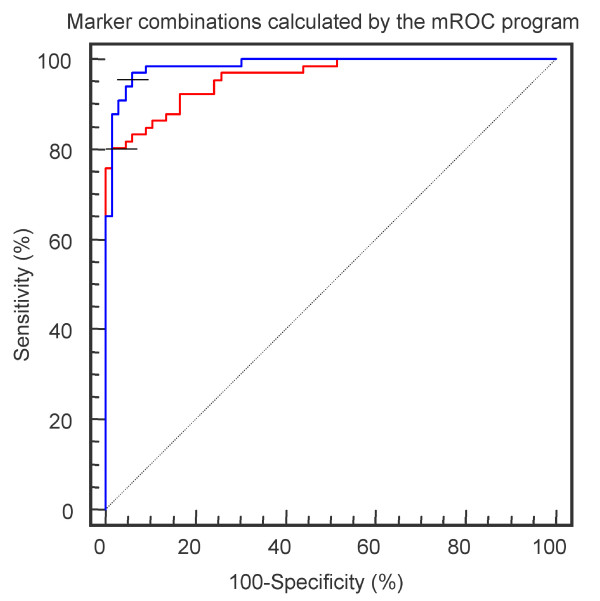
ROC curves for the best two-marker and three-marker combination calculated by the mROC program. The red line presents the ROC curve for the linear combination of MMP9 and TIMP1, the blue one the curve with the marker combination of MMP2, TIMP1, and MMP9. The equations of these virtual markers are given in Table 3.

ROC curves were generated to analyze the diagnostic values of the markers (Figures 1, 2). Table 3 presents the areas under the curve (AUC) as the overall index for the diagnostic performance of the respective single markers [31]. MMP2 was the marker with the highest AUC. To use the information of more than one marker, we also calculated the diagnostic performance of combinations of markers. From the practical point of view, we decided to use not more than three markers in combination. For that purpose, we used the ratio of the two best single markers (MMP2, TIMP1) and the best combinations with two and three markers calculated by the mROC program [29]. That special program calculates the linear combination, which maximizes the AUC for all markers selected and also for all two- and three-marker combinations among the selected markers. The equation for the respective combination is provided and can be used as a new virtual marker. The best two-marker combination included MMP9 and TIMP1, while the best three-marker combination included MMP2, MMP9, and TIMP1. The ROC curves of these three combinations and the corresponding AUCs in comparison to the best single marker MMP2 clearly show the distinct improvement of diagnostic performance if marker combinations were used (Table 3, Figures 1 and 2). As shown by the best two-marker combination, including MMP9 and TIMP1, the optimized combination does not always include the best single markers (Table 3). All data were statistically adjusted by applying the sequentially rejective Bonferroni method.

**Figure 3:**
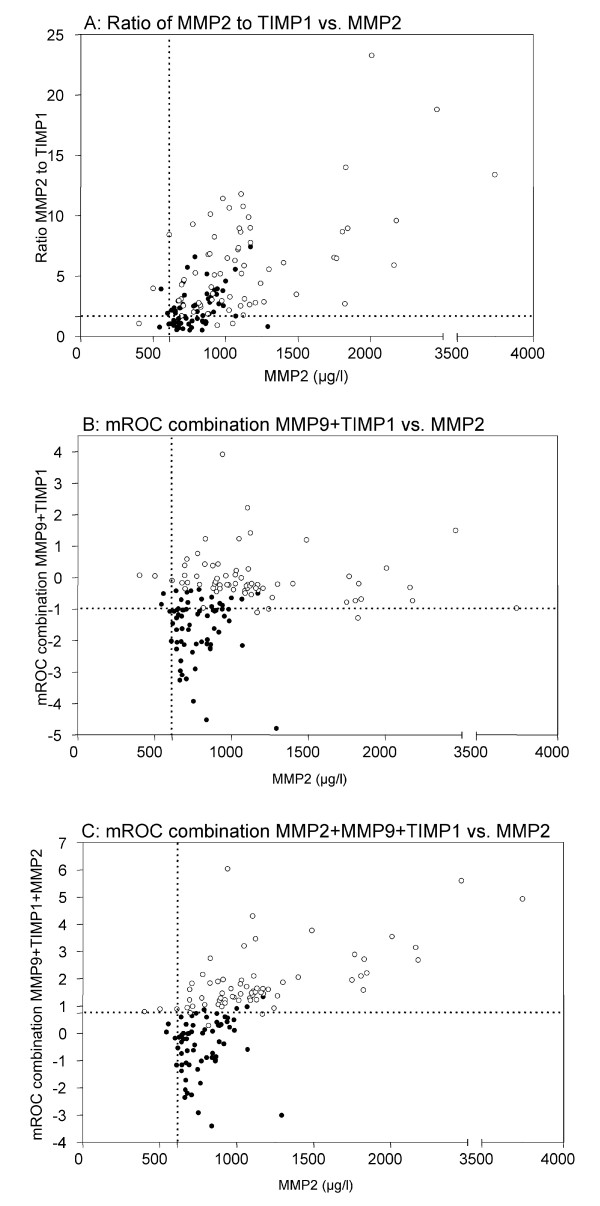
Dot-plots for presentation of the best marker combinations to distinguish between healthy persons (black dots) and bladder cancer patients (open dots). A: Ratio of MMP2 to TIMP1 versus MMP2; B: mROC combination of MMP9+TIMP1 versus MMP2; C: mROC combination of MMP2+MMP9+TIMP1 versus MMP2. The dashed lines indicate the cutoff values for 95% sensitivity of MMP2 and the corresponding marker combinations.

In addition to the overall result of diagnostic performance defined by the AUCs of the ROC analyzes, the sensitivity and specificity results of all markers and of the best two- and three-marker combinations were calculated at certain cutoffs. The marker concentrations with the highest diagnostic accuracy (minimal false-negative and false-positive results), marked in the ROC curves of Figures 1 and 2, were selected as cutoff values. Table 4 presents the diagnostic sensitivity and specificity of the MMPs and TIMPs to distinguish between healthy persons and bladder cancer patients. We included the cutoffs at the 95% limits of sensitivity and specificity and calculated the corresponding specificity and sensitivity values (Table 4). The improved sensitivity and specificity values are obvious if the two- and three-marker combinations calculated by the mROC program were used [29]. Both sensitivity and specificity increased to over 90% with the three-marker combination MMP2, MMP9, and TIMP1 (Table 4, Figure 2). These results could be confirmed by subsequent calculations with splitted data using 2/3 of samples for the calculation of the equations for the marker combinations by the mROC program and 1/3 of data as test samples (data not shown). For better visualization dot blots are shown for the best single marker MMP2 in combination with the marker combinations obtained by the mROC program with the highest sensitivities and specificities (Figure 3).

The positivity rates of the markers were additionally analyzed in the different groups of carcinoma patients. Based on the cutoff values given in Table 4, test results were classified as either negative or positive. These dichotomous variables were analyzed using the chi-square test for the association of sensitivity in relation to stage and grade categories, non-metastasized and metastasized bladder cancer. Plasma concentrations of MMP1, MMP3, MMP9, TIMP2, and the MTC1 did not correlate to tumour stage, grade, lymph node involvement, or distant metastasis. Only the sensitivity of TIMP1 was associated with the tumour grading (Chi-square, p = 0.038) as we expected from the results as presented in Table 2.

## Discussion

### The value of MMPs and TIMPs expression in bladder tissue

Despite the clinical significance on the pathogenetic impact of MMPs in human bladder cancer only a limited number of studies are available in the literature. MMP2, MMP9, and TIMP2 are the most frequently investigated metalloproteinases and inhibitors in human bladder tissue [4,32-34]. It has been described in the literature that elevated expressions of MMP2 and MMP9 in bladder cancer tissue at the mRNA and protein level are associated with advanced tumour stage, grade, and a decreased survival rate [32-34]. Kanayama et al. [33] found an elevated mRNA expression of MMP2 and MMP9 in tissue of muscular invasive bladder tumours compared to non-invasive tumours. The authors concluded that MMP2 and TIMP2 contribute to bladder tumour invasiveness and therefore might be useful prognostic markers in the future. Overexpressions of MMPs are associated with various pathological events [35]. Gakiopoulou et al. [36] found that TIMP2 is involved in regulation of apoptosis and is associated with an adverse prognosis in patients with TCC of the bladder. One group investigated MMP1 and MMP3 protein expression by immunohistochemistry [15]. They showed a correlation for MMP1 expression and tumour aggressiveness, but no detectable expression for MMP3. Vasala et al. [4] demonstrated an overexpression of MMP2 immunohistochemically. MMP2 overexpression correlated with bladder cancer stage, but not with grade. The 5-year survival rate for patients with bladder cancer was significantly lower in the MMP2 positive group.

In the most recent study on MMPs and their inhibitors urothelial carcinoma specimens were profiled for 24 MMPs and the four endogenous tissue inhibitors and their receptors using quantitative real time RT-PCR [37]. This study showed that MMP2, MT1-MMP, and MMP28 were very highly expressed in bladder tumour samples and MMP1, 7, 9, 11, 15, 19, and 23 were also highly expressed. TIMP1 and TIMP3 correlated with an increasing tumour grade. By laser capture microdissection of RNA it was possible to locate the MMP expression within the tissue. The study revealed that the most highly expressed MMPs are located in the stroma, except MMP13, which was located in the epithelium. Wallard et al. [37] confirmed in their study the impact of MMP1, MMP2, MMP9, and TIMP1 as potential diagnostic and therapeutic targets and supported the impact of those MMPs and TIMP1 as clinical markers for bladder cancer.

In neoplastic diseases an imbalance of MMPs and TIMPs, leading to an excess of degradative activity, is supposed to be linked to the invasive character of tumour cells [12,13]. Their proteolytic activity is activated by a complex cascade, which is not yet completely understood. Activated MMP1, MMP3 and latent forms of MMP2 and MMP9 are regulated and inhibited by endogenous proteins known as tissue inhibitors of metalloproteinase TIMP1 and TIMP2 [17]. Studies have shown that TIMP1 binds preferably to MMP9 and TIMP2 preferably to MMP2 [18]. Kugler et al. [38] stated for renal cell carcinoma (RCC) that the balance of MMP2- and MMP9- to TIMP1- and TIMP2-mRNA expression is a prognostic factor of tumour aggressiveness. Increased expression of TIMP1 in RCC correlates with poor prognostic variable including shortened patient survival. The paradoxical poor prognostic implication of TIMP overexpression documented in the literature complements the dual function of TIMPs and warrants further investigation [39].

### Diagnostic values of MMPs, TIMPs and their combination tested in blood plasma

There are only limited data available on circulating MMPs and TIMPs in patients with bladder cancer. Gohji et al. [40] analyzed MMP2 and MMP3 concentrations in blood serum of patients with bladder cancer. They found elevated concentrations of MMP2 and MMP3 in patients with advanced TCC of the bladder (pT2-T4, N+, M+) in comparison to the serum of patients with superficial tumors (Ta-T1, N0, M0). Their studies revealed further elevated MMP2, MMP3, and TIMP2 concentrations from patients after tumour resection, which might function as a predictive value for early detection of tumour recurrence [20,40]. Further, Guan et al. [16] correlated MMP9 expression in the serum to tumour grade and stage as a clinical prognostic factor. However, it has been shown that all MMP measurements in serum give equivocal values [23].

To our knowledge, there is no publication so far available on detection of MMPs and TIMPs in blood plasma for bladder cancer detection. On the basis of the concept that MMPs are synthesized in tissue and released into the blood stream we evaluated the levels of MMPs, their inhibitors, and MTC1 in blood plasma from patients with TCC of the bladder at different grades, stages, and with and without metastasis. Although citrate was recently suggested to be the most suitable anticoagulant to prepare blood samples for MMP measurements [41], comparable MMP activities in heparin and citrate plasma samples were found [42]. The plasma concentration of MMP2 was significantly higher in comparison to the control group whereas the concentrations of MMP1, TIMP1, TIMP2, and MTC1 were significantly lower from patients with non-metastasized TCC of the bladder in comparison to the healthy control group (Table I). In comparison to the expression data in tissue described above, these results confirm that circulating MMPs and TIMPs do not always reflect the direct tissue situation. That phenomenon, well known in clinical enzymology as enzyme distortion, has also to be considered for MMPs and TIMPs [43]. Since the mechanisms of release into extracellular space, the distribution in the intravascular compartment, and the elimination from that compartment can differently affect these analytes. Both changes of quantitative and qualitative relations between them are possible. For the complex interconnections between the various MMP components and pathways in tissue, Overall and Kleifeld [44] recently introduced the term "protease web" and underlined that the interactions between MMPs, TIMPs, and related components are more important than the single components. We believe that this complexity may essentially determine the distortion between tissue expression and blood pattern of MMPs and TIMPs. That aspect makes it also understandable that the levels of MMPs and TIMPs in plasma have a limited value to attribute these values to their expression in tissue and vice versa. Another important aspect is the fact that we only measured non-active MMPs. However, it is well known that, for example, the ratios of active to total MMP2 and MMP9, respectively are changed in tumour tissue and also in plasma [45,46].

Plasma concentrations from patients with metastasized tumours showed statistically significant higher values for TIMP1 compared to samples from non-metastasized tumours. That phenomenon could be a result of the dual function of the TIMP1 as already discussed in the previous chapter. Due to the possible pre-analytical interferences mentioned above it is not possible to compare our data measured in plasma to the data from investigations in serum. In this study the diagnostic performance criteria sensitivity, specificity, and ROC data revealed the best values for MMP2 as a separate tumour marker for TCC. Comparing our data on different MMPs MMP2 was proved to be the best marker alone. It was statistical significantly elevated in superficially, invasive bladder tumours and in metastasized bladder tumours in comparison to the healthy control group (p < 0.001) (Table 1). MMP2 was also shown to stand alone as clinical tissue marker in other studies as described above.

Using the statistical mROC program we have calculated the different sensitivities and specificities for all MMPs and TIMPs at their different cutoff points (Figures 1, 2 and Table 4) and additionally determined the best combination of two and three markers (Table 4, Figure 3). The best single indicators with the highest sensitivity and specificity (MMP2, TIMP1) did not necessarily reached the highest sensitivities and specificities in combination with the next best indicators. However, MMP2 does not reach a satisfying sensitivity when tested alone. At the cutoff point it reaches only 75% sensitivity and specificity than when tested in combination with MMP9 and TIMP1 (97% sensitivity, 94% specificity). Our results show that different indicators in specific combinations could result in an improved sensitivity and specificity in comparison to the best single indicator test by itself, which has never been shown so far in the literature (Table 3, 4, Figure 2). We presented in this study that single markers have only a limited diagnostic value. Separate analyzes of MMP1, MMP2, MMP3, MMP9, TIMP1, TIMP2, and MTC1 in plasma do not allow a prognostic prediction for tumour grading, staging, or metastasis, but certain combinations could be very helpful. Although the limitations of our study should be considered that the results are based on a small number of patients and patients of benign urological diseases (inflammations) were not included. We suggest specific MMPs and their combinations as potential helpful diagnostic indicators in early and advanced bladder cancer. To some degree there might exist an overfitting in the resulting data of the two virtual mROC classifiers because MMP9 and TIMP1 in the two best combinations are the markers with the highest correlation to each other.

There are controversial opinions in the literature. However, we used a similar approach as Piironen et al. [47], who included covariates of a correlation coefficient <0.7 into a common multivariate model. Further prospective studies should prove the usefulness of these marker combinations. However, so far it is not economic efficient and not practical to combine those three markers for routine tumour screening. Further investigations are still necessary for easier analyzes of MMPs and TIMPs and a more economical application to the clinical routine.

## Conclusion

Improved detection methods for diagnosis of asymptomatic bladder cancer are essential for an early and reliable diagnosis and a curative treatment of this tumour entity. The standard diagnostic is still the cystoscopy with biopsy of suspicious bladder tissue, which is, however, invasive. Several research projects focus on finding tumour markers, which are easy and inexpensive to obtain, for example in urine and blood, for a non-invasive way of bladder cancer detection.

The values of MMPs and TIMPs as potential diagnostic tumour markers in bladder cancer have been discussed in the literature. In earlier studies expression changes of MMPs and their inhibitors have been detected in tumour tissue and in blood serum. But for the first time we could evaluate that MMP2 as a statistically significant marker in blood plasma for bladder cancer detection with an increased diagnostic value in combination with MMP9 and TIMP1. We found that the markers with the highest diagnostic values do not reach the sensitivity and specificity like other markers would show in a combination. The clinical value of separately used MMPs and TIMPs as tumour markers in blood plasma is limited. On the basis of these observations we conclude that measurements of plasma MMP and TIMP levels in relevant combinations may provide important data for selecting patients for treatment with drugs that interfere with MMP and TIMP activities.

## Abbreviations

Area under the curves, AUC; Non-metastasized Bladder carcinoma, BCa; Extracellular matrix, ECM; Sandwich enzyme-linked immunosorbent assay, ELISA; Metastasized bladder carcinoma, mBCa; Matrix metalloproteinase, MMP; MMP1/TIMP1-complex, MTC1; Receiver operatoring characteristic, ROC; Transitional cell carcinoma of the bladder, TCC; Tissue inhibitor of matrix metalloproteinase, TIMP; Transurethral resection of the bladder, TURB.

## Competing interests

The author(s) declare that they have no competing interests.

## Authors' contributions

AS-has made contribution to concept, design, interpretation of data, statistical calculations, and carried out drafting and writing the manuscript;

SB-has made contributions to analysis and collecting of data, carried out molecular studies (e.g. ELISA tests);

DS-has made substantial contribution to acquisition of data (e.g. patient's data collection from charts collection of patient's blood plasma);

SL-has been involved in revising critically the manuscript, collected data from the Department of Pathology;

KJ- has been involved in drafting the manuscript, has given final approval of the version to be published, was involved in statistical data analysis and carrying out research experiments.

All authors read and approved the final manuscript.

## Pre-publication history

The pre-publication history for this paper can be accessed here:



## Figures and Tables

**Table 1:** Plasma concentrations of MMP1, MMP2, MMP3, MMP9, TIMP1, TIMP2, the MMP1/TIMP1-complex (MTC1), and the ratio of MMP2/TIMP1 of healthy volunteers, of patients with non-metastasized (BCa) and metastasized (mBCa) TCC of the bladder.

	**Controls (n = 79)**	**BCa (n = 57)**	**mBCa (n = 11)**
**MMP1 (μg/l)**	5.6 (0.6–24.4)	2.8 (0.6–20.6) *p < 0.001	4.4 (0.6–24.4) *p > 0.05 ^+^*p > 0.05*
**MMP2 (μg/l)**	749 (547–1295)	1030 (406–3751) *p < 0.001	1114 (820–2182) *p < 0.001 ^+^*p > 0.05*
**MMP3 (μg/l)**	11.9 (0.18–34.9)	9.9 (0.3–37.4) *p > 0.05	17.7 (0.6–27.9) *p > 0.05 ^+^*p > 0.05*
**MMP9 (μg/l)**	19.4 (3.52–411)	22.9 (4.4–412) *p > 0.05	56.3 (6.1–269) *p > 0.05 ^+^*p > 0.05*
**TIMP1 (μg/l)**	423 (121–1776)	184 (57.8–1084) *p < 0.001	300 (207–1029) *p > 0.05 ^+^*p > 0.05*
**TIMP2 (μg/l)**	146 (91–374)	132 (24.9–424) *p < 0.01	128 (76.0–335) *p > 0.05 ^+^*p > 0.05*
**MTC1 (μg/l)**	30.5 (4.1–143)	24.8 (2.8–63.3) *p > 0.05	30.3 (15.1–74.3) *p > 0.05 ^+^*p > 0.05*
**Ratio of MMP2/TIMP1**	1.87 (0.47–7.37)	5.83 (0.87–23.3) *p < 0.05	3.07 (1.02–9.56) *p < 0.001 ^+^*p > 0.05*

Key: TCC = transitional cell carcinoma of the bladder; MMP = matrix metalloproteinase; TIMP = tissue inhibitor of matrix metalloproteinase; MTC1 = MMP1/TIMP1-complex. Values are presented as median values with ranges (in parentheses). Statistical differences among the groups were calculated by the non-parametric Kruskal-Wallis test. Statistical significances of at least p < 0.05 are typed in '**bold**'; comparisons are indicated by following symbols: *, between controls and BCa, as well as mBCa; +, between BCa and mBCa.

**Table 2:** Correlation coefficients according to Spearman between MMPs, TIMPs, and to stage and grade for all patients suffering from bladder cancer and healthy controls.

	**MMP1**	**MMP2**	**MMP3**	**MMP9**	**TIMP1**	**TIMP2**	**MTC1**
**T-Stage**	0.05	0.15	0.16	0.10	0.32*	0.05	0.12
**Grading**	-0.10	0.14	0.07	0.12	0.29*	0.01	0.11
**MMP1**	1.00	-0.30*	0.08	0.16	0.44*	0.10	0.47*
**MMP2**		1.00	0.11	-0.06	-0.26*	0.13	0.02
**MMP3**			1.00	0.01	0.16	0.08	0.09
**MMP9**				1.00	0.61*	0.14	0.081
**TIMP1**					1.00	0.33*	0.26*
**TIMP2**						1.00	0.06
**MTC1**							1.00

*Significances of at least p <0.05.

**Table 3:** Areas under the ROC curves (AUC) for MMPs and TIMPs as single markers and for marker combinations in comparison to MMP2 as the single marker with the highest AUC^a^.

**Variables**	**AUC^a ^**	**P values**^**b**^	
	(95% confidence interval)	Comparison with AUC 0.5	Pairwise AUC comparison with the AUC for MMP2
MMP1	0.72 (0.64 – 0.80)	<0.001	0.249
MMP2	0.82 (0.74 – 0.88)	<0.001	
MMP3	0.54 (0.45 – 0.63)	<0.443	<0.001
MMP9	0.62 (0.53 – 0.70)	<0.054	<0.001
TIMP1	0.78 (0.70 – 0.84)	<0.001	0.443
TIMP2	0.65 (0.56 – 0.73)	0.004	0.005
MTC1	0.61 (0.52 – 0.69)	0.086	0.005
Ratio of MMP2/TIMP1	0.87 (0.79 – 0.92)	<0.0001	0.254
MMP9 + TIMP1^c ^(mROC combination)	0.96 (0.91 – 0.99)	<0.001	<0.001
MMP9 + TIMP1 + MMP2^c ^(mROC combination)	0.99 (0.95 – 1.00)	<0.001	<0.001

^a^AUCs were calculated with all bladder carcinoma patients and healthy controls.

^b^P values were adjusted according to the sequential Bonferroni correction [29].

^c^The equations for the marker combinations calculated by the mROC program were Z = 0.0204 MMP9 - 0.00415 TIMP1 for the best two marker combination, and Z = 0.00159 MMP2 + 0.0227 MMP9 - 0.00446 TIMP1 for the best three marker combination, respectively. Using these equations a new virtual marker was calculated.

**Table 4:** Diagnostic sensitivities and specificities of MMPs, TIMPs, and the MMP1/TIMP1-complex (MTC1) as single markers and two- and three-marker combinations to distinguish between healthy persons and bladder cancer patients at the 95% limits of sensitivity and specificity, respectively.^a^

**Marker (μg/l)**	**Sensitivity (%)**^**b**^	**Specificity (%)**
Single marker		
MMP1		
16.5	95 (85 – 98)	4.2 (1 – 12)
0.95	24 (15 – 34)	95 (85 – 98)
MMP2		
613	95 (85 – 98)	7 (2 – 15)
1071	47 (35 – 60)	95 (85 – 98)
MMP3		
25.2	95 (85 – 98)	3 (0.4 – 10)
3.1	13 (6 – 24)	95 (85 – 98)
MMP9		
6.0	95 (85 – 98)	20 (11 – 31)
106	12 (5 – 22)	95 (85 – 98)
TIMP1		
655	95 (85 – 98)	26 (16 – 38)
158	38 (27 – 51)	95 (85 – 98)
TIMP2		
315	95 (85 – 98)	1.4 (0.2 – 7.4)
112	40 (28 – 52)	95 (85 – 98)
MTC1		
57.3	95 (85 – 98)	12 (6 – 22)
6.9	6 (2 – 15)	95 (85 – 98)
		
Marker combinations^c^		
Ratio of MMP2 to TIMP1		
1.65	95 (85 – 98)	46 (34 – 58)
5.45	49 (36 – 61)	95 (85 – 98)
mROC combinations^d^		
MMP9 + TIMP1		
-1.004	95 (85 – 98)	74 (62 – 84)
-0.482	82 (70 – 90)	95 (85 – 98)
MMP9 + TIMP1 + MMP2		
0.745	95 (85 – 98)	94 (85 – 98)
0.841	94 (85 – 98)	95 (85 – 98)

^a^Data results from ROC analysis (see Figures 1, 2) performed with the 79 controls and 68 patients with bladder cancer. The cutoffs correspond to the values at 95% sensitivity and specificity as indicated.

^b^95% confidence intervals are given in parentheses.

^c^As marker combinations the ratio of the two best single markers (MMP2, TIMP1) and the best combinations based on the highest AUCs with two and three markers calculated by the mROC program (see Table 2; further details see text) were selected.

^d^The equations for the marker combinations used for the calculation of the respective new virtual marker is given in the legend of Table 3.
